# An Accurate Representation of the Number of bZIP Transcription Factors in the *Triticum aestivum* (Wheat) Genome and the Regulation of Functional Genes during Salt Stress

**DOI:** 10.3390/cimb46050268

**Published:** 2024-05-07

**Authors:** Xin Liu, Selvakumar Sukumaran, Esteri Viitanen, Nupur Naik, Sameer Hassan, Henrik Aronsson

**Affiliations:** 1 Department of Biological and Environmental Sciences, University of Gothenburg, Box 461, 405 30 Gothenburg, Sweden; xinliu0508@outlook.com (X.L.); a10selsu@gmail.com (S.S.); esteri.viitanen@bioenv.gu.se (E.V.); nupurnaik9@gmail.com (N.N.); sameerhassan1@gmail.com (S.H.); 2 Triticeae Research Institute, Sichuan Agricultural University, Wenjiang 611130, China

**Keywords:** BARI Gom-25, bZIP, salinity, salt stress, transcription factor, wheat

## Abstract

Climate change is dramatically increasing the overall area of saline soils around the world, which is increasing by approximately two million hectares each year. Soil salinity decreases crop yields and, thereby, makes farming less profitable, potentially causing increased poverty and hunger in many areas. A solution to this problem is increasing the salt tolerance of crop plants. Transcription factors (TFs) within crop plants represent a key to understanding salt tolerance, as these proteins play important roles in the regulation of functional genes linked to salt stress. The basic leucine zipper (bZIP) TF has a well-documented role in the regulation of salt tolerance. To better understand how bZIP TFs are linked to salt tolerance, we performed a genome-wide analysis in wheat using the Chinese spring wheat genome, which has been assembled by the International Wheat Genome Sequencing Consortium. We identified 89 additional bZIP gene sequences, which brings the total of bZIP gene sequences in wheat to 237. The majority of these 237 sequences included a single bZIP protein domain; however, different combinations of five other domains also exist. The bZIP proteins are divided into ten subfamily groups. Using an in silico analysis, we identified five bZIP genes (*ABF2*, *ABF4*, *ABI5*, *EMBP1*, and *VIP1*) that were involved in regulating salt stress. By scrutinizing the binding properties to the 2000 bp upstream region, we identified putative functional genes under the regulation of these TFs. Expression analyses of plant tissue that had been treated with or without 100 mM NaCl revealed variable patterns between the TFs and functional genes. For example, an increased expression of *ABF4* was correlated with an increased expression of the corresponding functional genes in both root and shoot tissues, whereas *VIP1* downregulation in root tissues strongly decreased the expression of two functional genes. Identifying strategies to sustain the expression of the functional genes described in this study could enhance wheat’s salt tolerance.

## 1. Introduction

*Triticum aestivum* (wheat) is one of the most widely cultivated crops in the world, with bread wheat contributing to 95% of total wheat production. Wheat provides 19% of daily protein and 18% of daily calories to 7.7 billion people worldwide [1]. In addition to being a major source of starch and energy, wheat also provides adequate amounts of various beneficial compounds, e.g., vitamins (notably, B vitamins), minerals, dietary fiber, and phytochemicals [2]. Although wheat consumption is increasing on a global level, the challenges posed by phenomena such as increased soil salinity have resulted in reduced yields, affected the global economy, and increased the threat of social unrest [3]. Salinity affects more than 6% of land worldwide and is one of the most severe abiotic stresses for crop plant productivity [4]. Salt stress induces a complex series of deleterious effects on plants, ultimately resulting in growth inhibition, disturbances in mineral nutrition, alterations in membrane permeability and cellular osmotic balance, the generation of oxidative stress via reactive oxygen species (ROS), and the inhibition of enzymatic activity [5].

Salt stress in plants can be divided into two phases that they must withstand [6]. The first phase, the osmotic phase, is associated with salt-affected soil, which inhibits water uptake by roots. This triggers a signal from the roots to the shoots, prompting stomatal closure to prevent water loss through transpiration. Consequently, reduced CO_2_ influx occurs due to stomatal closure, leading to a decrease in photosynthetic activity, plant development, and, ultimately, the final yield [7,8].

The subsequent phase involves the excess accumulation of Na^+^ and Cl^−^ ions in the shoot, originating from the salt-affected soil via root absorption. To prevent cellular toxicity, these ions must be excluded from the cell. This can be achieved through limiting the root uptake or root-to-shoot transport of Na^+^ and Cl^−^. Another strategy involves sequestering Na^+^ and Cl^−^ ions into vacuolar compartments while accumulating osmotically balancing solutes in the cytoplasm to mitigate the toxicity of Na^+^ and Cl^−^ [7,8].

Nevertheless, certain plants can thrive even when subjected to different types of abiotic and biotic stresses, such as drought, salinity, and extreme temperatures, that otherwise adversely affect plant growth, development, and crop yields. This indicates that plants have evolved an array of stress responses that are necessary for adapting to variable environments, which will become more pronounced as climate change intensifies [9,10]. Transcription factors (TFs) play crucial roles in almost all biological processes, and interact with specific cis-regulatory sequences in the promoter regions of stress-responsive genes to regulate expression and, subsequently, improve stress tolerance. The bZIP TF family is one of the largest and most conserved gene families among eukaryotic organisms. Members of this family are involved in regulating developmental and physiological processes, including photomorphogenesis, leaf and seed formation, energy homeostasis, and abiotic and biotic stress responses [11,12,13,14]. Members have a highly conserved bZIP domain composed of 60–80 amino acids that contains two functional regions: a basic region and a leucine zipper (ZIP). The basic region preferentially binds to DNA sequences with an ACGT core, such as G-box (CACGTG), C-box (GACGTC), and A-box sequences (TACGTA) [15], whereas the leucine zipper region is associated with dimerization specificity based on the presence of hydrophobic and non-polar residues [16]. These two regions are linked by a hinge region [17,18].

A heptad repeat of a repetitive pattern of seven amino acids, labelled a to g, is crucial for the dimerization of bZIP to occur. Amino acids at positions a and d (Leu or other hydrophobic amino acids) are key players in forming a hydrophobic interface at the protein’s C-terminal end [19]. This process creates an amphipathic α-helix, essential for bringing together two bZIP monomers to form homo- or heterodimers [20,21]. The formation of these dimers is not random among individual bZIPs [19,22]. The stability and specificity of this dimerization is dependent on the amino acid composition at the leucine zipper region [23]. This suggests that the dimerization of bZIP proteins plays a highly specific role in gene regulation, as it is vital for bZIPs’ ability to bind to DNA [19,20].

A large number of bZIPs have been identified in plant genomes, e.g., 75 in *Arabidopsis thaliana* (Arabidopsis) [24], 131 in *Glycine max* (soybean) [25], 89 in *Oryza sativa* (rice) [26], 92 in *Sorghum bicolor* (great millet) [27], and 187 in *Triticum aestivum* (wheat) [28]. Phylogenetic analysis has revealed that bZIP homologues in angiosperms can be divided into 13 subgroups, and many bZIP members from subgroups A, C, and S have been reported to participate in plant abiotic stress responses [28,29,30]. Sequence analyses and chromosomal distribution has revealed that the bZIP TF family has evolved via gene duplication [26].

Great efforts have been made to sequence and annotate the complex hexaploid wheat genome [31,32,33,34,35,36,37]; recently, a draft sequence of the 15.8 Gb hexaploid wheat genome was generated by sequencing isolated chromosome arms [38]. This represents an important milestone for our understanding of wheat genetics, and may prove to be a crucial resource for the identification of gene expression and co-expression networks during developmental stages. Considering that wheat is exposed to detrimental salinity stress in various areas around the world, the most recently sequenced wheat genome can be used to better understand how bZIP proteins are related to salinity stress. This not only enhances the accuracy of the number of bZIPs present but also facilitates the identification of novel ones and their unique characteristics and expression patterns, thereby facilitating the understanding of bZIPs’ role in abiotic stress. This insight can then be used to improve agricultural research and breeding programs.

## 2. Materials and Methods

### 2.1. Identification of bZIP Sequences

The Hidden Markov Model (HMM) file of the wheat domain (PF00249) was downloaded from the PFAM database to identify bZIP sequences in the wheat genome (PFAM version 32.0). To match the bZIP sequences with the wheat genome, the proteome of the wheat cultivar Chinese spring was downloaded from the Ensemble plants database (https://ftp.ensemblgenomes.ebi.ac.uk/pub/plants/release-41/fasta/triticum_aestivum/pep/, accessed on 17 March 2020) and used as a reference. The HMM profile of the bZIP domain was used as a query to scan the wheat proteome using HMMER software (Version 3.1) with an E value of 1 × 10^−5^. An in-house Python script was used to extract amino acid sequences of the bZIP-domain-containing proteins found in the wheat proteome. Redundant protein sequences were identified using CD-HIT (https://github.com/weizhongli/cdhit-web-server, accessed on 10 May 2020) with a sequence identity cut-off of 100%, and representative sequences were used for further analysis.

### 2.2. Transcription Factor Binding Site Prediction

The upstream regions (2000 bp) of all the identified genes were extracted from the IWGSC Chinese spring wheat genome, which was accessed via the Ensembl database. The Bedtools getfasta option was used to extract the upstream sequences of individual genes. The bZIP protein sequences were subjected to profile inference tool analysis to identify the JASPAR TF-binding profiles. An in-house Python script (https://github.com/Sameerpython/Transcription-Factors, accessed on 17 June 2020) was written to search the JASPAR database. The identified bZIP TF factor profiles were in silico analyzed to verify expression patterns upon salt stress by using the dataset available at the Wheatomics1.0 website (http://202.194.139.32/, accessed on 15 November 2021), which included two different wheat cultivars exposed to salt stress under the “wheat biotic” options. Next, a Position Weight Matrix (PWM) of the five most prominent bZIP sequences (*ABF2*, *ABF4*, *ABI5*, *EMBP1*, and *VIP1*) was downloaded in MEME format from the JASPAR CORE database. The upstream region of every gene was then scanned against the four PWMs to predict the bZIP transcription binding site using the FIMO tool (http://meme-suite.org/index.html, accessed on 25 February 2022), with a *p*-value < 1 × 10^−5^.

### 2.3. Functional Gene Extraction

The 144 genes that were previously identified to be involved in salt stress [36] were run against the 3000+ target genes from FIMO analysis (Section 2.2 above) for each of the ABF2, ABF4, ABI5, EMBP1, and VIP1 TF families. More specifically, the 144 genes were extracted based on gene IDs from FIMO analysis using R, and potential functional salt-stress-related genes were identified. Furthermore, the identified bZIP functional genes were in silico analyzed to verify expression patterns upon salt stress by using the dataset available at Wheatomics1.0 website (http://202.194.139.32/, accessed on 15 November 2021), which contains two different wheat cultivars exposed to salt stress under the “wheat biotic” options.

### 2.4. Phylogenetic Analysis

The bZIP sequences were aligned with the bZIP HMM profile using the HMMAlign module in HMMER to obtain a more accurate alignment of the distant homologues. The aligned sequences were then used to build a phylogenetic tree using MEGA7 (Molecular Evolutionary Genetics Analysis, version 7.0) software [39]. Before tree construction, the best model for maximum-likelihood analysis was determined using MEGA7. A bootstrap of 100 replicates was used when building the phylogenetic tree, which was constructed using representative bZIP sequences that had been identified through the total bZIP profile search.

### 2.5. Principal Component Analysis of bZIP Sequences

The R package peptides [40] was used to calculate 66 amino acid descriptors for the 20 amino acids in the bZIP domain sequences selected for this study. A Principal Component Analysis (PCA) was conducted to extract the most significant features from the feature vectors (1320 features) generated by the R package peptides. PCA was chosen because this statistical technique reduces multidimensionality, decreases the number of dimensions, and can derive patterns from the dataset. For the analysis, the data inputs were organized in a matrix denoted as X, which was composed of N and K dimensions, with N representing the number of sequences (observations) and K representing the number of features (variables). In PCA, the principal components, i.e., K-dimensional space, which explain the greatest amount of variance contained within the dataset are defined. The orientation of the model plane in the K-dimensional variable space was influenced by the loadings, which quantify the contributions of each of the original variables to the principal components. The principal components were the eigenvectors of the covariance matrix of the data matrix X, and were, thus, orthogonal. The largest eigenvalues corresponded to the dimension that explained the larger degree of variation in the dataset.

### 2.6. bZIP Protein Structure Analysis

Both proteins and DNA molecules can undergo conformational changes in their structure to form functional complexes. To understand the conformational difference between the free crystal structures of bZIP and its bound state with DNA, crystal structures of various bZIP proteins, when bound to DNA, were downloaded from PDB. To compare protein folding and active site dynamics between apo and holo structures of bZIP proteins, RMSD and RMSF data were extracted and analyzed using Bio3D R package (https://pubmed.ncbi.nlm.nih.gov/32734663/, accessed on 20 March 2022).

### 2.7. Plant Material, Growth Conditions, and Treatments

BARI Gom-25, a moderately salt tolerant wheat variety developed by the Bangladesh Agriculture Research Institute (BARI), used previously for salt tolerance assays, was used for the study [41,42,43]. Seeds were germinated on wet filter paper (Munktell-filter paper—A1-100-80^TM^) for three days in the dark at room temperature. At the beginning of the fourth day, seedlings were transferred to a hydroponic growth system. The hydroponic growth system contained tap water mixed with Nelson Garden Hydroponic Nutrition^TM^ (2 mL/L), and included continuous aeration. After six days of hydroponic growth, 100 mM NaCl was added to one unit of the system to induce salt stress, whereas another unit of the system was run without salt stress to serve as a control. After six days with or without salt stress, the shoots and roots were harvested separately (2–3 h after onset of light), snap frozen in liquid nitrogen, and stored at −80 °C until further analyses. The root and shoot samples were collected from a pool of 20 different plants.

### 2.8. Quantitative Real-Time PCR (qPCR)

Frozen plant shoot and root tissues were pulverised using a Mixer Mill MM 301 (Retsch GmBH, Haan, Germany) at 2 × 15 s. Total RNA was extracted from the tissues according to the NucleoSpin RNA Plant^TM^ kit instructions (Macherey-Nagel, Düren, Germany). The iScriptTM cDNA Synthesis Kit (Bio-Rad, Hercules, CA, USA) was used to synthesize cDNA from a total of 100 ng of root or shoot RNA. The qPCR analyses were carried out on *a* BioRad CFX96 Real Time^TM^ system following the instructions from the SsoAdvanced Universal SYBR Green Supermix^TM^ (Bio-Rad) kit for qPCR. The wheat actin gene was chosen as the housekeeping gene. The data were analyzed using the 2^–ΔΔCt^ method. Each qPCR run included three biological replicates, which were all subjected to three technical replicates. Primers for the genes of interest (Supplementary Table S1) were designed using the Primer3 website. The qPCR assays were run using 12-day-old plant shoot and root tissues of wheat lines grown with or without salt stress in a hydroponic setup.

## 3. Results

### 3.1. Identification of bZIP Transcription Factors in the Wheat Genome

To comprehensively identify the bZIP genes of interest in *Triticum aestivum* (wheat), a Hidden Markov Model (HMM) profile of the bZIP domain (PF00170) was used to search the latest genome database of wheat. As a result, a total of 258 bZIP gene sequences were characterized, which encoded 541 transcript isoform sequences. After the removal of partial sequences, a total of 237 putative sequences remained. As such, our analysis yielded 89 new putative sequences. To further confirm the high degree of sequence conservation among bZIP family members, the conserved domain—which is a shared feature of bZIP family members—was used in the search. The length of the bZIP domain ranged between 78 to 83 amino acids. However, the full length of the protein varied from 273 to 291 amino acids (https://ftp.ensemblgenomes.ebi.ac.uk/pub/plants/release-57/fasta/triticum_aestivum/pep/Triticum_aestivum.IWGSC.pep.all.fa.gz, accessed on 10 November 2022).

### 3.2. Domain Architecture of bZIP Transcription Factors

**Figure 1:**
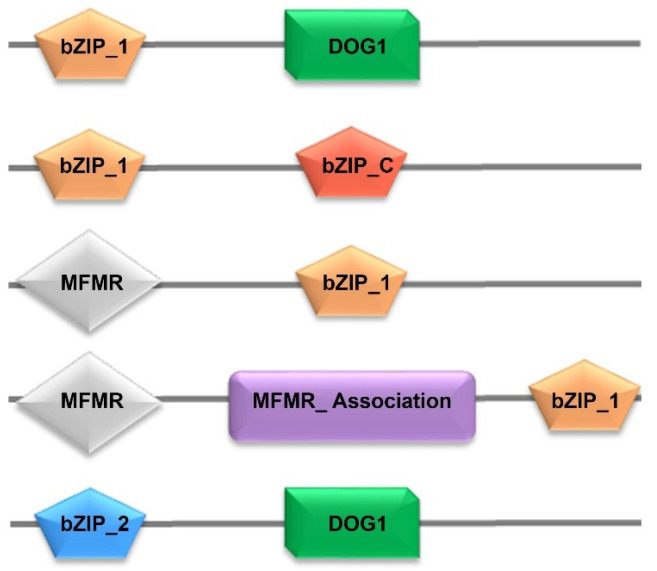
A total of five distinct domain architectures were observed among the bZIPs identified from the Chinese spring wheat genome. The numbers represent each individual architecture. The five distinct architectures involved a total of six different individual domains, namely, bZIP_1: basic leucine zipper domain 1 (PF00170); bZIP_2: basic leucine zipper domain 2 (PF07716); bZIP_C: basic leucine zipper domain C (PF12498); DOG1: delay of germination 1 (PF14144); MFMR: multifunctional mosaic region (PF07777), and MFMR_assoc: multifunctional mosaic region association (PF16596).

The functions of proteins can often be inferred from their domain architecture. A PFAM analysis of the 258 identified bZIPs returned 195 protein sequences with one bZIP_1 domain and 63 protein sequences with multiple domains, including on bZIP_1 (Figure 1). The identified bZIPs demonstrated five distinct domain architectures which involved six different domains (Figure 1). More specifically, 38 sequences included a single bZIP_1 domain together with DOG1, eight sequences had bZIP_1 and bZIP_C domains, seven sequences had bZIP_1 and MFRM domains, and nine sequences involved bZIP_1, MFMR, and MFMR_assoc domains. Finally, there was one instance in which the domain architecture of a bZIP involved a single bZIP_2 together with DOG1 (Figure 1). The analysis of domain architecture yielded an intriguing discovery: the identification of the DOG1 domain. The DOG1 domain (Delay in Germination) serves as a regulatory protein for maintaining primary seed dormancy in plants. During salt stress, seeds experience a reduction in water absorption ability, leading to an ionic imbalance that, ultimately, inhibits seed germination [44].

### 3.3. Phylogenetic Analysis of bZIP Transcription Factors

**Figure 2:**
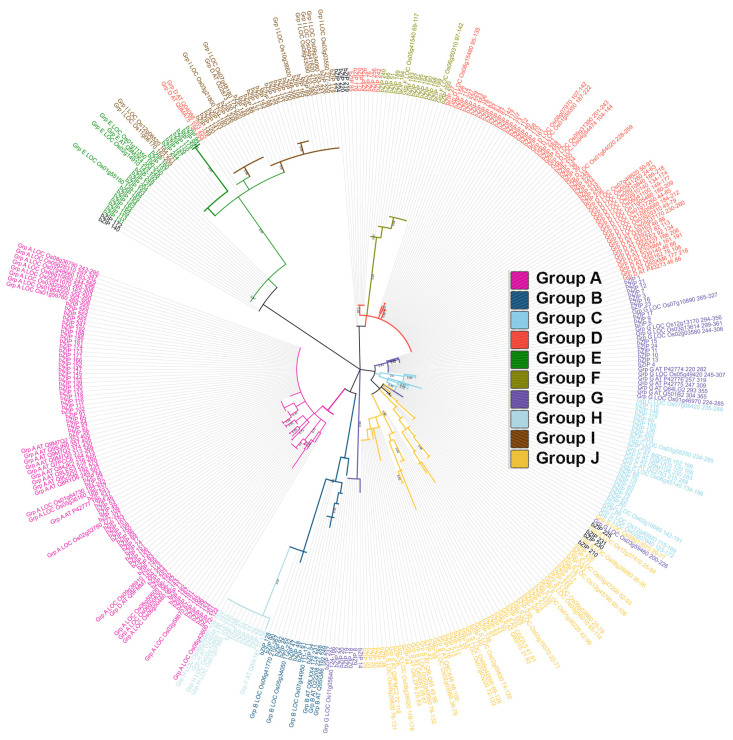
Phylogenetic analysis of the bZIP_1 protein sequences from the *Triticum aestivum* (wheat), *Oryza sativa* (rice), and *Arabidopsis thaliana* (Arabidopsis) genomes. The evolutionary history of the 258, 83, and 45 protein sequences in the wheat, rice, and Arabidopsis genomes (Supplementary Table S2), respectively, was inferred using the maximum-likelihood statistical method, which revealed ten groups of subfamilies. Group A: pink; Group B: blue; Group C: sky blue; Group D: red; Group E: green; Group F: olive green; Group G: purple; Group H: light blue; Group I: brown; and Group S: yellow. A bootstrap of 100 replicates was used to infer the evolutionary history of the bZIP_1 protein sequences.

To understand the phylogenetic and evolutionary relationships between the bZIP TFs in wheat, other known bZIP TFs from species such as Arabidopsis and rice were included in an unrooted Neighbor-Joining tree with 100 bootstraps (Figure 2). The analysis revealed 258 bZIP TFs, which were assigned into ten subfamilies along with the orthologous bZIP TFs from Arabidopsis and rice.

Based on the previously reported classification of bZIP TFs in Arabidopsis and rice [29], the sequences were pre-labelled with the respective group names and combined with the newly identified bZIP TF sequences for wheat. The clustering yielded ten groups, viz., A, B, C, D, E, F, G, H, I, and S. The sequences with known groups [29] clustered together to form independent groups. The groups A, B, C, D, E, F, G, H, I, and S include 59, 15, 18, 45, 13, 13, 16, 7, 32, and 40 sequences, respectively. In the phylogenetic tree, the groups A, H, and E branched into clades with high bootstrap values of 70, 97, and 96, respectively.

### 3.4. Principal Component Analysis

**Figure 3:**
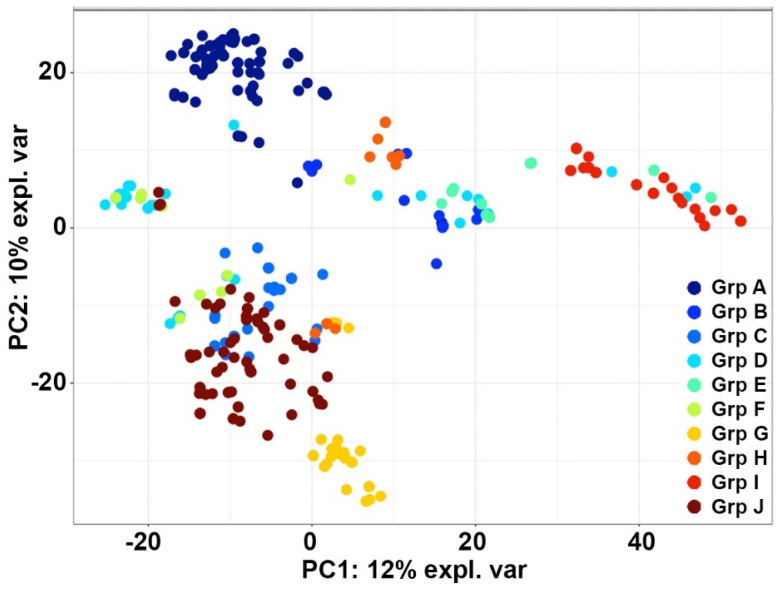
Principal component analysis of bZIP_1 protein sequences. According to the Principal Component Analysis, the 258 bZIP_1 protein sequences were grouped into ten subfamily groups, namely, group A (Grp A), group B (Grp B), group C (Grp C), group D (Grp D), group E (Grp E), group F (Grp F), group G (Grp G), group H (Grp H), group I (Grp I), and group J (Grp J), which are shown in different colors as described in the legend.

To further validate the clustering of the bZIP TF sequences, a PCA analysis using 1320 features for the 258 sequences was performed. Group A and G sequences were clearly separate from other groups and formed clear clusters, whereas group I sequences showed some degree of similarity with groups C, D, E, and F (Figure 3). Groups S and C predominantly clustered together, while three sequences from group G showed a similar clustering pattern as sequences from group H (Figure 3). Principal component 1 (PC1) contained variables which explained 12% of the variance in the dataset, while the variables in principal component 2 (PC2) explained 10% of total variance in the data.

### 3.5. Structural Analysis of bZIP Proteins

**Figure 4:**
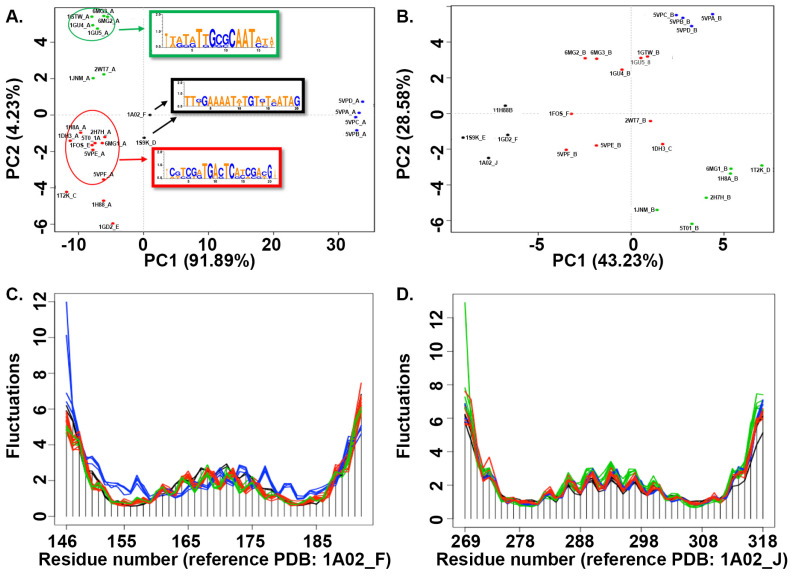
Conformational changes in chains A and B from 26 experimentally determined structures of bZIP. (**A**). Structures representing chain A, projected onto the first two principal components (PCs). Each point corresponds to one specific structure; holo structures are presented in black, green, or red according to which kind of DNA sequence it is bound to, while apo structures are presented in blue. (**B**). Structures representing chain B, projected onto the first two principal components (PCs); each colored dot represents one specific structure, with the same color-coding as in (**A**). Residue-wise fluctuation analysis of chains A and B of the collected ensemble of bZIP structures (**C**,**D**).

We identified 34 bZIP crystal structures in the PDB database; of these, 24 structures involved two helixes binding to both strands of DNA, designated as chain A and chain B, respectively. These 24 structures were subjected to further analysis to gain more insight into the conformational differences among the structures. The clustering of structures based on RMSD values yielded four different clusters. Of these, one cluster contained four apo structures of bZIP that demonstrated clear conformational differences when compared to the other bZIP holo structures, all of which were bound to DNA (Figure 4A). Furthermore, the DNA-bound bZIP structures could be separated into three different clusters, which revealed that the structures have distinct conformational differences when bound to DNA (Figure 4A). An assessment of the conformations of both chains A and B in the analyzed structures revealed certain conformational changes, as shown in the PCA plot (Figure 4A,B). The structures representing chain A formed close clusters, whereas the structures representing chain B were more dispersed within the clusters (Figure 4A,B). The residual fluctuations in chain A structures were greater than what was observed for chain B structures among the different clusters (Figure 4C,D). Across both chains, all of the structures exhibited large fluctuations at the two termini of their sequences. However, the four apo structures of chain A (blue cluster) showed clearly larger fluctuations in the N-termini than in the C-termini, which was not observed for the other three clusters (Figure 4C). The conformational fluctuations between the apo and DNA-bound structures were not as distinct for chain B structures, with the apo structures showing similar fluctuations as the structures belonging to the red cluster (Figure 4D). A comparison of the apo structures (blue cluster) against the three other DNA-bound clusters (black, green, and red) made it clear that the amino acid residues between positions 149 to 184 have distinct conformations among the different clusters of apo structures (Figure 4A). All of these structures demonstrated fluctuations of over 3 Å in the helix between amino acid residues 161–175 (Figure 4A).

A comparison of the DNA sequences of binding sites for structures within different clusters revealed that structures which bound to similar DNA sequences were closely grouped in the PCA space (Figure 4A). For example, five structures of the green cluster (1GU5, 1GTW, 1GU4, 6MG2, and 6MG3) were bound to DNA sequences with a conserved G-[GC]-G-C-A-A-T region, whereas the other two structures of the green cluster, 1JNM and 2WT7, were bound to different DNA sequences. The two structures (1A02 and 1S9K) of the black cluster bound to DNA sequences that were 80% identical. Among the ten structures included in the red cluster, five structures (5T01, 1FOS, 2H7H, 5VPE, and 5VPF) that were grouped closely in the PCA space were found to bind to DNA sequences that had a conserved [TC]-[CG]-[GT]-[AG]-T-G-A-[CG]-T-C-[AG] region. Interestingly, two structures of the red cluster (1H8A and 1H88) which had 100% identical amino acid sequences and bound to the same DNA binding site nevertheless existed far apart in the PCA space. Structure 6MG1 was found to bind to a conserved T-[GT]-G-C-G-C-A-A-T region of DNA. The remaining two structures included in the red cluster, 1GD2 and 1T2K, each bind to different DNA sequences, and did not group closely with other members of the red cluster in the PCA space.

### 3.6. DNA Binding Site Analysis

**Figure 5:**
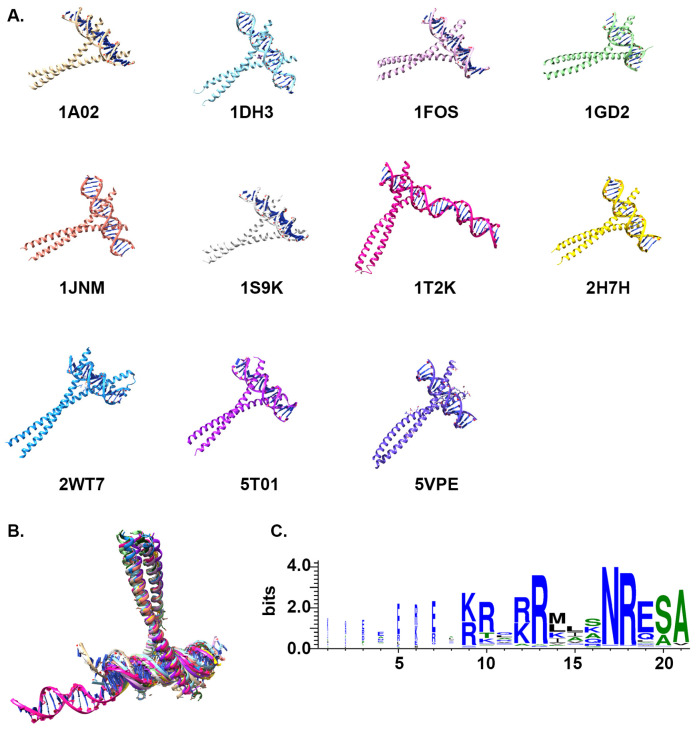
DNA binding motif of bZIP_1. (**A**). Interactions between 11 bZIP_1 structures and DNA (1A02, grey; 1DH3, light blue; 1FOS, light purple; 1GD2, light green; 1JNM, orange; 1S9K, white; 1T2K, pink; 2H7H, yellow; 2WT7, blue; 5T01, purple; and 5VPE, medium blue). (**B**). A superimposed image showing how the 11 structures bind to DNA. (**C**). A WebLogo representation of which amino acid residues of the 258 identified bZIP_1 domain sequences bind to DNA.

The bZIP TFs contain a highly conserved ribonucleotide reductase (RNR) domain. A search of the PBD database uncovered a total of 11 distinct structures for the bZIP_1 domain (Figure 5A,B). Of these eleven structures (Figure 5), 5VPE was found to bind to eight ligands of 1,2-ethanediol (EDO*8) and two ligands of phosphate (PO4*2). In addition, the PBD database revealed that five of the structures had metal co-factors. Of the 11 structures, one (1T2K) was selected for further studies concerning protein–DNA interactions. The nucleotide sequence of the DNA binding site for 1T2K was identified using the PDBsum database, and was mapped onto the 258 previously identified bZIP sequences (Figure 5C).

### 3.7. bZIP Family Gene Expression under Salt Stress

The previous in silico analyses of bZIP family members based on available online RNA sequencing data also showed that certain bZIP genes are expressed during salt stress, namely, ABF2, ABF4, ABI5, EMBP1, and VIP1. Thus, all these bZIP proteins have DNA-binding transcription factor activity (Supplementary Table S3). Moreover, target genes that were predicted to be regulated by these bZIP genes were also scrutinized for their responsivity to salt stress before selecting them for further expression studies.

#### 3.7.1. ABF2 Family

**Figure 6:**
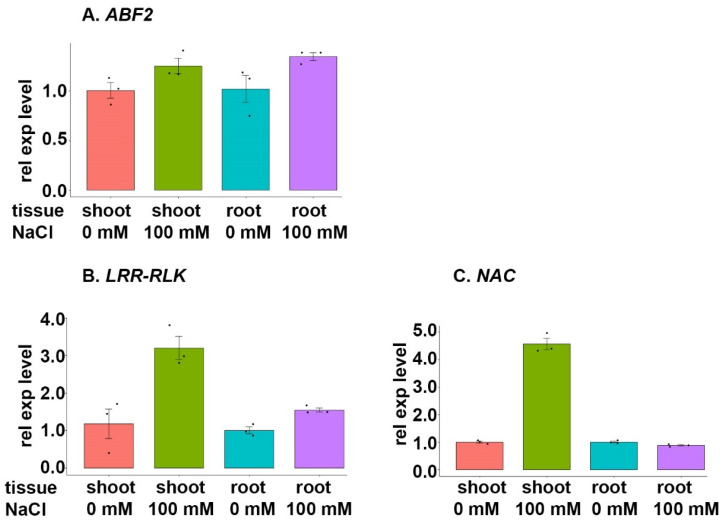
Expression analysis of the gene encoding TF *ABF2* and selected target genes during salt stress. BARI Gom-25 wheat seedlings were grown on a hydroponic system for six days, after which the seedlings were treated with or without 100 mM NaCl for another six days. Shoot and root tissues were then subjected to expression analysis of the TF *ABF2* (**A**), and the target genes *LRR-RLK* (leucine-rich repeat-receptor-like kinase) (**B**) and *NAC* (**C**).

The gene encoding the *ABF2* TF (TraesCS5D02G244500) was upregulated in both shoot and root tissue samples that had been subjected to salt stress (Figure 6A). The corresponding functional gene, *ERL_gD* (TraesCS7D02G060400), also known as a *leucine-rich repeat-receptor-like kinase (LRR-RLK),* was also upregulated in both shoot and root tissues under NaCl stress (Figure 6B). The second functional gene regulated by the *ABF2* TF, which is the gene encoding the *NAC* TF (TraesCS3B02G439600), was upregulated in shoot tissues and slightly downregulated in root tissues during salt stress (Figure 6C).

#### 3.7.2. ABF4 Family

**Figure 7:**
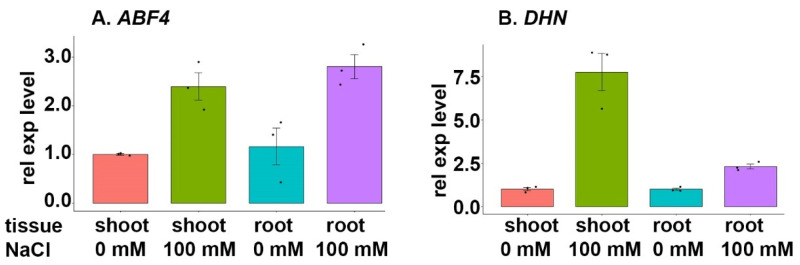
Expression analysis of the gene encoding TF *ABF4* and a selected target gene during salt stress. BARI Gom-25 wheat seedlings were grown on a hydroponic system for six days, and then treated with or without 100 mM NaCl for another six days. Shoot and root tissues were then subjected to expression analysis of the TF *ABF4* (**A**), and the target gene *DHN* (dehydrin) (**B**).

The gene encoding *ABF4* TF (TraesCS5A02G237200), a bZIP-domain-containing protein with a function in the abscisic-acid-activated signalling pathway, was upregulated in both shoot and root tissue samples (relative to control samples) exposed to NaCl stress (Figure 7A). The corresponding functional gene, *DHN* (dehydrin) (TraesCS6D02G333600), displayed strong upregulation in the shoot tissues, but only slight upregulation in the root tissues, during salt stress (Figure 7B).

#### 3.7.3. ABI5 Family

**Figure 8:**
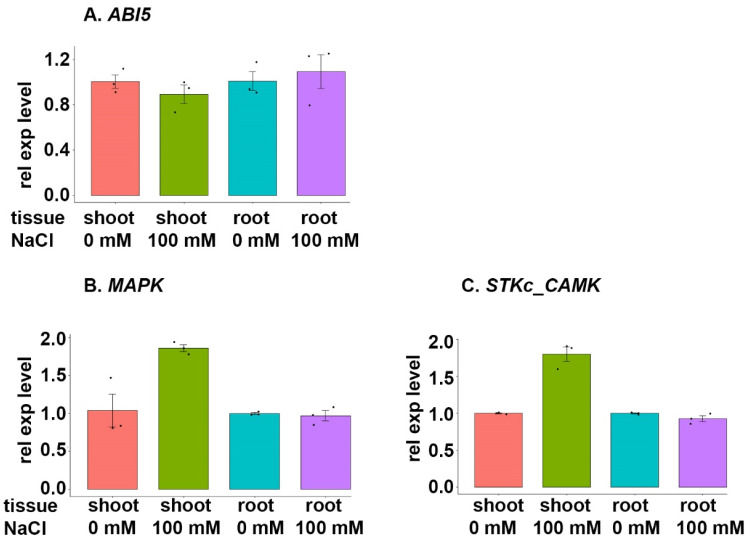
Expression analysis of the gene encoding TF *ABFI5* and selected target genes during salt stress. BARI Gom-25 wheat seedlings were grown on a hydroponic system for six days, after which the seedlings were treated with or without 100 mM NaCl for another six days. Shoot and root tissues were then subjected to expression analysis of the TF *ABF4* (**A**), and the target genes *MAPK* (mitogen-activated protein kinase) (**B**) and *STKc_CAMK* (calcium-dependent protein kinase) (**C**).

The gene encoding *ABI5* TF (TraesCS1A02G306300) demonstrated a slight downregulation in shoot tissues and upregulation in root tissues when plants were exposed to NaCl stress (Figure 8A). The functional genes regulated by *ABI5*, a *mitogen-activated protein kinase (MAPK)* (TraesCS3A02G242100) and a *calcium-dependent protein kinase (STKc_CAMK)* (TraesCS2A02G456100), were upregulated in shoot tissues during NaCl stress (Figure 8B,C). The expression of these functional genes in the root tissue samples from plants exposed to salt stress did not differ from what was observed for control plants (Figure 8B,C).

#### 3.7.4. EMBP1 Family

**Figure 9:**
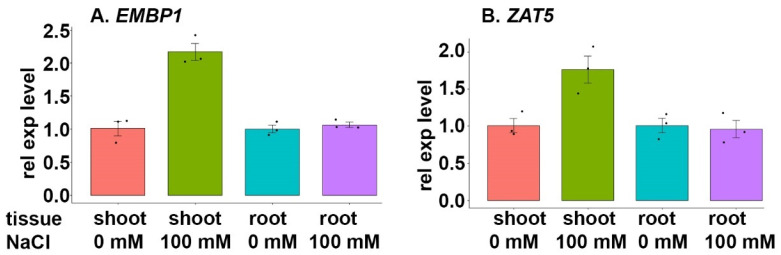
Expression analysis of the gene encoding TF *EMBP1* and a selected target gene. BARI Gom-25 wheat seedlings were grown on a hydroponic system for six days, after which the seedlings were treated with or without 100 mM NaCl for another six days. Shoot and root tissues were then subjected to expression analysis of the TF *EMBP1* (**A**), and the target gene *ZAT5* (**B**).

The qPCR results showed that the gene encoding *EMBP1* TF, a *histone-specific transcription factor (HBP1)* (TraesCS2B02G269600), was upregulated in shoot tissue samples but similarly regulated in root tissue samples with or without salt stress (Figure 9A). The corresponding functional gene, which encodes *zinc finger protein ZAT5* (TraesCS5B02G490600), showed similar expression patterns during salt stress; more specifically, shoot tissue samples revealed an upregulated expression during salt stress, while root tissue samples demonstrated a similar level of expression during salt stress (Figure 9B).

#### 3.7.5. VIP1 Family

**Figure 10:**
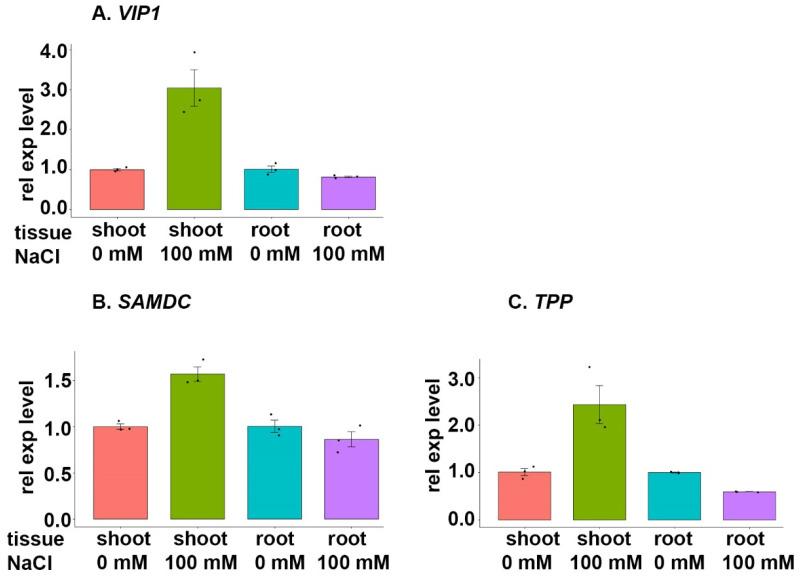
Expression analysis of the TF *VIP1* and selected target genes during salt stress. BARI Gom-25 wheat seedlings were grown on a hydroponic system for six days, after which the seedlings were treated with or without 100 mM NaCl for another six days. Shoot and root tissues were then subjected to expression analysis of the TF *VIP1* (**A**), and the target genes *SAMDC* (S-adenosylmethionine decarboxylase proenzyme) (**B**) and *TPP* (trehalose 6-phosphate phosphatase) (**C**).

The expression of the gene encoding *VIP1* (TraesCS5B02G124200) was upregulated in shoot tissues, yet slightly downregulated in root tissues, during NaCl stress (Figure 10A). The corresponding functional gene regulated by *VIP1*, which encodes the *S-adenosylmethionine decarboxylase proenzyme* (*SAMDC*) (TraesCS2B02G372900), was upregulated in shoot tissues and slightly downregulated in root tissues during NaCl stress (Figure 10B). The second functional gene regulated by *VIP1*, which encodes *trehalose 6-phosphate phosphatase (TPP)* (TraesCS3A02G289300), was upregulated in shoot tissues and downregulated in root tissues during NaCl stress (Figure 10C).

## 4. Discussion

Based on HMM information of the *bZIP* domain (PF00170), we were able to characterize a total of 258 *bZIP* TF genes in wheat. Thus, the number of *bZIP* genes reported in this study clearly exceeds previous reports, i.e., 197 and 227 wheat *bZIP* genes [45,46]. After further analyzing the *bZIP* TF sequences, we were able to assign these genes, along with orthologous *bZIPs* from Arabidopsis and rice, into ten subfamilies, which is in accordance with previous data [28]. A recent report which clarified the phylogeny of sesame classified the bZIP TF family into nine subfamilies [47]. Similarly, the bZIP TF family members identified for cucumber were divided into six subfamilies [48]. Comparing the crystal structures of bZIP proteins from various biological sources offered an insight into the conformational changes upon binding to DNA molecules. This indicates that bZIP proteins can undergo shifts during their functional stages, which has implications for understanding their binding to the cis-elements of target genes, such as those involved in responding to salt stress.

As expected, most of the predetermined groups clustered together; however, some groups also showed separate clustering patterns. Given the complexity and large size (ca. 16 Gb) of the hexaploid wheat genome—when compared, for example, to the diploid genomes of Arabidopsis and rice—a certain degree of functional diversity in the target *bZIPs* was expected. Therefore, the large number of TF subfamilies in wheat may be attributed to its hexaploid genome.

Our results reveal that the domain architecture of certain bZIPs in wheat includes the DOG1 domain together with the bZIP domains; this has been previously reported in maize [49]. The subgroup-A bZIP proteins may hold great promise for further studies, as members show the largest share of conserved motifs, which may be closely linked to the abiotic stress responses of wheat, Arabidopsis, and rice [50]. In previous studies conducted on Arabidopsis and rice, genes belonging to subgroup A of the bZIP family were found to play significant roles in plants subjected to various abiotic stresses, particularly drought stress associated with ABA signalling [50]. All five identified TFs linked to salt stress, except for EMBP1, belong to subgroup A (Figure 2). Since both drought and salt stress involve the use of ABA for stomatal closure, they may share similar mechanisms [51]. An analysis of the 11 distinct bZIP structures determined to be homologous to wheat genes (Table 1) revealed that a total of 21 amino acid residues interact with the DNA binding site (Figure 5C).

### 4.1. bZIP Expression under Salt Stress

The performed in silico analyses revealed that certain bZIP TF genes, namely, *ABF2*, *ABF4*, *ABI5*, *EMBP1,* and *VIP1,* contribute to the regulation of salt stress. For instance, the increased expression of *ABF2* was correlated with the increased expression of target genes in both root and shoot tissue samples (Figure 6). Thus, the upregulation of this TF strengthens the salt tolerance. However, other bZIP TFs, along with the target genes, were also found to be downregulated following treatment with 100 mM NaCl. Notably, *VIP1* was downregulated in root tissue, and this subsequently induced a strong downregulation of two target genes in root tissue (Figure 10). It is important to note that TFs, i.e., *ABF2* and *ABF4*, and target genes found to be downregulated might follow a diurnal expression pattern; i.e., they might have lower expression levels at the sampling time and higher expression levels later in the day. Alternatively, these genes could exhibit a strong but temporary expression at the onset of salt stress, i.e., within the first hours. Therefore, additional assays are necessary to rule out or validate these potential scenarios.

### 4.2. ABF2 Family

The gene encoding *ABF2* was upregulated in BARI Gom-25 shoot and root tissues that had been exposed to NaCl stress (Figure 6A). This supports previous findings from different wheat varieties [42,43,44,45,46,47,48,49,50,51,52,53,54]. Moreover, the ABF2 expression increases under conditions of hyperosmotic salinity, salt sensitivity, and salt stress [55].

The functional genes under *ABF2* control, i.e., *leucine-rich repeat-receptor-like kinase* (*LRR-RLK*) and *NAC* TF, mainly showed an upregulated expression during salt stress in the shoot tissue samples from BARI Gom-25 seedlings (Figure 6B,C). Given that the expression of ABF2 is linked with the expression of other TFs, e.g., NAC, and the potential of these two TFs largely influence the expression of downstream genes, these results were expected and highlight how important bZIPs are to the plant response to salt stress.

Previous studies in rice that focused on a homolog of the target gene *ERL_gD*, i.e., *SIK1* (salt-inducible kinase), found an increased expression of *OsSIK1* (a gene which encodes stress-induced protein kinase) in plants that were exposed to NaCl stress [56]. The research found that *OsSIK1* expression is mainly induced by salt stress and drought, both conditions which lead to enhanced activities of antioxidant enzymes, such as peroxidase, superoxide dismutase, and catalase [56]. Interestingly, transgenic rice plants with *OsSIK1* overexpression showed a stronger tolerance to NaCl and drought stress than control, wild-type plants [56,57,58], whereas the knock-out mutants *sik1-1* and *sik1-2*, along with RNA interference (RNAi) plants, were sensitive to NaCl and drought stress [56]. The activities of the three enzymes mentioned above were unaffected in the knock-out mutants under normal conditions, but demonstrated some extent of downregulation under salt stress [56]. The results suggest that *SIK1* may promote salt tolerance by increasing the expression of antioxidative enzymes. This is in accordance with the downregulation of ROS scavenging enzymes, which leads to a hypersensitivity to oxidative stress responses [56,59,60,61]. Given that *SIK1* influences the expression of genes that are linked to an increased ROS scavenging and antioxidative capability under NaCl stress, it can be concluded that *ERL_gD* plays an important role in salt tolerance.

### 4.3. ABF4 Family

The gene encoding the *ABF4* TF was upregulated in BARI Gom-25 shoot and root tissues during NaCl stress (Figure 7A). The expression of *ABF4*, which encodes a bZIP-domain-containing protein with a role in the abscisic-acid-activated signalling pathway, induced upregulation in a corresponding functional gene, *DHN* (dehydrin), under NaCl stress.

Dehydrins, also known as group II LEA proteins, have been extensively studied in different species under various abiotic stresses. Homologs of the target *DHN* have been previously reported to improve salt tolerance in a somatic hybrid between bread wheat cultivar JN177 and *Thinopyrum ponticum* (tall wheatgrass), *Capsicum annuum* L. (pepper), Arabidopsis, and *Musa* spp. cv. *Karibale Monthan* (banana), among others [62,63,64,65]. It has also been shown that ABA and NaCl treatment substantially upregulate the expression of *TaDHN1*, *TaDHN2,* and *TaDHN3*, all of which are homologs of *DHN*; this strongly suggests that dehydrins may contribute to a high salt tolerance in wheat [65]. Interestingly, Qin and Qin (2016) found that *TaDHN1*, *TaDHN2*, and *TaDHN3* expression was initially strongly upregulated in the roots, with this upregulation occurring noticeably later in the leaves. The fact that roots are the first plant tissue to encounter soil salinity could explain the observations that the expression of target genes was staged. In this study, *ABF4*, which participates in the ABA signalling pathway, together with the target gene *DHN*, both showed a noticeable upregulation during NaCl stress. This indicates that dehydrins could be upregulated in an ABA-dependent stress-signalling pathway to improve salt tolerance.

### 4.4. ABI5 Family

Our results indicated that, while the expression of *ABI5* decreased in shoot tissue during NaCl stress, the expression levels of the corresponding functional genes increased in shoot tissue during the same salt stress period (Figure 8). These functional genes were identified to encode a *mitogen-activated protein kinase* (*MAPK*) and a *calcium-dependent protein kinase* (*STKc_CAMK*).

MAPK cascades are an essential part of plant growth and development, along with responses to abiotic stresses [66]. This type of plant signalling was previously studied using transgenic Arabidopsis plants overexpressing ZmSIMK1 (*Zea mays* salt-induced mitogen-activated protein kinase 1), which is a homolog of MAPK [66]. *ZmSIMK1* was strongly induced by NaCl stress, and improved the salt tolerance of the transgenic Arabidopsis. Other studies have also shown that MAPKs play an important role in plant salt tolerance [66,67,68,69]. The MAPK cascade is known to be modulated through transcriptional programming, and MAPKs subsequently phosphorylate specific effector proteins, leading to the activation of cellular responses [70]. Putative MAPK receptors are highly specific for external or intracellular stimuli, which could explain the upregulation of the target *MAPK* gene (TraesCS3A02G242100) in wheat shoot tissues under NaCl stress [70,71,72]. Furthermore, Ca^2+^ ions act as vital secondary messengers for signal transduction in plant defence mechanisms, and this raises the question of whether calcium-dependent protein kinases, specifically *STKc_CAMK* (TraesCS2A02G456100), are upregulated in parallel with *MAPK* (TraesCS3A02G242100) in wheat shoot tissues under NaCl stress to induce a stronger stimulus and enhance salt tolerance.

### 4.5. EMPB1 Family

The expression of *EMPB1*, which encodes a histone-specific TF (*HBP1*), was upregulated in shoot tissues, with no major change in root tissues during NaCl stress (Figure 9A). *HBP1* has been implicated in seed maturation, seed coat color, seed dormancy, germination rate, and root length [55,73,74]. Prior studies of the *HBP1* expression in wheat have revealed tissue-specific patterns, with the highest levels observed in the grain [55,75,76]. The target gene of *HBP1*, which encodes *zinc finger protein ZAT5*, has been shown to respond to hyperosmotic salinity [55].

*ZAT12* has been identified to be a homolog of *ZAT5* in Arabidopsis [77]. Earlier studies in Arabidopsis have shown that plant C2H2-zinc finger proteins that possess an EAR motif [L/FDLNL/F(x)P], including Arabidopsis ZAT12, are important for regulating different defense responses against several abiotic and biotic stresses [77,78,79]. The characterized EAR motif, which is located in the C-terminal of various C2H2-zinc finger proteins, plays an essential role in the active repression of transcription [77,79]. Although the role of stress-associated EAR-repressors needs further investigation, it has been speculated that they may play a role in controlling the initiation of a stress-activated gene expression under different abiotic stresses, such as salt [79]. In the present study, we found that the *HBP1* expression increases in shoot tissues but stayed unchanged in root tissues upon salt stress (Figure 9A). *ZAT5*, which is regulated by *HBP1*, showed a similar expression pattern as the TF upon NaCl treatment (Figure 9B). This transcriptional behavior could be explained as if the expression of *ZAT5* is stimulated by the TF, thereby starting to act as an active repressor via an EAR motif; as such, the activation of *ZAT5* expression upon salt stress could stimulate the repression of further downstream genes to, for example, withstand salt stress.

### 4.6. VIP1 Family

An upregulated *VIP1* expression was witnessed in BARI Gom-25 shoot tissues during salt stress, while a slight downregulation was observed in root tissues upon NaCl treatment (Figure 10A). The expression pattern of *VIP1*, which encodes a bZIP-domain-containing TF, under salt stress held true for the corresponding functional genes, *SAMDC* and *TPP* (Figure 10B,C).

A previous study in *Brassica napus* L. (canola) roots focused on the biosynthesis of trehalose and the correlation to NaCl stress, with several genes identified to play a key role in the regulation of salt tolerance [80]. In the same study, a homolog of *TPP* which encodes alpha,alpha-trehalose-phosphate synthase was downregulated in canola roots upon NaCl stress [80]. Similar trends were observed in wheat root tissue samples analyzed in this study. Trehalose, a common disaccharide, plays an important role in regulating sugar metabolism in plants, especially under stress conditions [81]. Based on the presented results, TPP, which catalyzes the dephosphorylation of trehalose-6-phosphate to trehalose, is negatively affected by NaCl stress in wheat roots. However, the upregulation of the TF *VIP1*, a bZIP-domain-containing protein (TF), in wheat shoot tissue under NaCl stress was found to induce the upregulation of *TPP*, which indicates that *VIP1* activates *TPP* transcription under NaCl stress.

Based on our findings, trehalose metabolism is negatively affected by NaCl stress in wheat roots, but simultaneously upregulated in shoots; this is indicative of a possible shift in plant signalling and trehalose metabolism during the plant stress response.

The other target gene, *SAMDC*, which encodes the S-adenosylmethionine decarboxylase proenzyme, is involved in polyamine (PA) biosynthesis and demonstrated a similar regulation as *TPP* by the *VIP1* TF. PAs are small molecules that function as secondary messengers in signalling pathways; as such, it is unsurprising that the levels of these molecules frequently increase during stress [82]. *SAMDC* demonstrated a notable upregulation in wheat shoots upon NaCl stress, and similar results have been reported in wheat during drought stress [83]. It has also been reported that—in *Vigna radiata* L. (mung beans)—increased PA levels are associated with enhanced salt tolerance [84].

The expression analysis unveiled a notable upregulation of bZIP transcription factors—*ABF4*, *EMBP1*, and *VIP1*—under salt stress. This discovery underscores the potential involvement of these genes in mediating salt stress responses within the wheat genome. A further exploration of the specific functions and regulatory mechanisms of these bZIP genes could deepen our comprehension of wheat’s adaptation to salinity stress. Consequently, this research holds significant promise for enhancing crop resilience in harsh salt environments. Regulating the regulator, bZIP, through changes in affinity for dimerization and DNA binding represents an important advancement. This can be achieved via the gene editing of crucial sites, coupled with molecular modeling and simulations. Such approaches can lead to more accurate predictions regarding which target sites will actually contribute to an improved stress tolerance [19,24,85].

For improving abiotic stress tolerance, e.g., salt tolerance, in agriculture, an essential molecular strategy is altering the gene regulation to activate or inhibit them in response to stress [19,86]. Thus, editing transcription factors emerges as crucial, e.g., bZIP linked to salt tolerance [87]. Since the amino acid composition within the leucin zipper plays an important role for the diversification of the hetero- and homodimer formed among different bZIPs, they serve as a major target for modification, capable of affecting the stability and specificity of the resulting dimers [85]. While the direct editing of the bZIP itself is obvious, targeting its promoter, as well as the promoters of its target genes, remains viable as well. Nonetheless, validating the functionality of individual bZIPs is important before selecting candidates for the editing approach, i.e., to ensure no detrimental effect on plant development and yield as, for example, functional redundancy might occur.

## 5. Conclusions

The bZIP TF family plays a crucial role in abscisic acid (ABA) signalling when plants are exposed to abiotic stress, such as drought and salinity [18]. An earlier study previously identified 187 *bZIP* genes in wheat, and classified these genes into ten subfamilies [28]. However, it turns out that previous reports significantly underestimated the actual number of *bZIP* genes within the wheat genome. We identified 258 bZIP TF proteins in the recently published wheat genome based on the HMM profile of the bZIP domain. Based on the sequences presented in a recent analysis of the diversity of wheat bZIPs, we created an HMM profile that was specific for bZIPs in the wheat genome; this may have helped identify novel *bZIP* genes in wheat. Plant bZIPs rely on various mechanisms to influence the transcription of target genes, such as DNA binding, the ability to form homo- and heterodimers, and interactions with proteins that do not contain a leucine zipper. This diversity among bZIPs suggests that these proteins could be involved in a variety of stress responses, with the evolution of different members of the bZIP TF family driving the adaptability of plants.

A greater knowledge of bZIP TF members, including domain architectures and binding specificity, could provide a deeper understanding of how various stress responses are regulated in plants. Understanding the molecular network of bZIP TFs as well as the specific role in salt tolerance could lead to the development of salt-tolerant wheat varieties that could adapt to the increasing area of saline agricultural land.

## Figures and Tables

**Table 1:** All of the eleven bZIP protein names, including the DNA binding site along with the organism sampled during crystallization.

IDs	Protein Name	Organism	DNA Binding Site
1A02	NFAT, FOS AND JUN	*Homo sapiens*	ATTTGTTTC
1DH3	CREB bZIP-CRE COMPLEX	*Mus musculus*	TGACGTC
1FOS	C-FOS:C-JUN	*Homo sapiens*	AGTC
1GD2	bZIP TRANSCRIPTION FACTOR PAP1	*Schizosaccharomyces pombe* (strain 972/ATCC 24843)	AGGTTACGTAA
1JNM	Jun/CRE Complex	*Homo sapiens*	TCGATGA
1S9K	Human NFAT1 and Fos-Jun	*Homo sapiens*	ATATGTGTA
1T2K	RF3, ATF-2 and Jun	*Homo sapiens*	AAATGAC
2H7H	JUN bZIP homodimer	Avian sarcoma virus (strain 17)	CGATGAC
2WT7	MafB:cFos	Synthetic construct, *Mus musculus*	ATTGCTGAC
5T01	Human c-Jun	Synthetic construct, *Homo sapiens*	CTATGA
5VPE	FosB/JunD bZIP	*Homo sapiens*	GGTGACTC

## Data Availability

The data that support the findings of this study are available from the corresponding author upon reasonable request.

## Supplementary Materials

The following supporting information can be downloaded at: https://www.mdpi.com/article/10.3390/cimb46050268/s1, Table S1: Primers for the qPCR expression analyses. Table S2: The phylogentic tree was constructed using 258, 83, and 45 protein sequences in the wheat, rice, and Arabidopsis genome, respectively. Table S3: bZIP transcription factor proteins—proposed functionalities, processes, and cellular components based on Gene Ontology results.
