# Supplementary material for: An Accurate Representation of the Number of bZIP Transcription Factors in the Triticum aestivum (Wheat) Genome and the Regulation of Functional Genes during Salt Stress
Source: Curr Issues Mol Biol. 2024 May 7;46(5):4417–36. doi: 10.3390/cimb46050268 (PMC11120151; doi:10.3390/cimb46050268)
Supplement: Supplementary file 1 [file cimb-46-00268-s001.zip › cimb-2942271-supplementary.pdf]

Supplementary Table S1. Primers for the qPCR expression analyses

bZIP transcription factor primers and actin primers			
**bZIP TF**	**Gene**	**Forward Primer (5'-3')**	**Reverse Primer (5'-3')**
ABF2	TraesCS5D02G244500	GGACCTGAACGAGGAACTGG	CCGTCCGAACTGATCCTTCA
ABF4	TraesCS5A02G237200	ACTGGTGAGGAAACAGGCAG	CCGTCCGAACTGATCCTTCA
ABI5	TraesCS1A02G306300	GAGCGCATTGGTTACAGCAG	TGGCTGAGGACCCAAATGAC
EMBP-1	TraesCS2B02G269600	TGAGAAGGGCAGTTCTGCTG	TCTCGTGTCTGAGGACTCGT
VIP1	TraesCS5B02G124200	GCCACTACGCTATCAGCACA	CTCCATGGACTGCAACCGAA
Actin	TraesCS1A02G274400	TCTATTTTGGCCTCTCTTAGCAC	TTTCCTGTACCCCTTATTCCTC

bZIP target gene primers			
**bZIP TF**	**Target gene**	**Forward Primer (5'-3')**	**Reverse Primer (5'-3')**
ABF2	TraesCS7D02G060400	CCATCCCTTCCACACTGTCC	CTTGGGATGTCGCCTGTGAG
	TraesCS3B02G439600	ATCATGCACGAGTACCGCC	CACAGCACCCAGTCATCCAA
ABF4	TraesCS6D02G333600	ACGAGAGAGGAGCACAAGGC	CCCATGCCATCATCCTCAGAC
ABI5	TraesCS3A02G242100	CCCCAAGGATAGACCAACGG	GGCAGGATGGTTCTCTCTCT
	TraesCS2A02G456100	ATTTCGGCCTCTCCGTCTTC	CAGCACTTCTGGGGCTACAT
EMBP-1	TraesCS5B02G490600	CATCAAGGAGCGGAAGCGT	CATCTCGAACCCCAGCCC
VIP1	TraesCS2B02G372900	ATGCTCCTGCTCACCATTCC	TCCCACGGGAGTACTTCACA
	TraesCS3A02G289300	CGTGTCCGAAGCTGGGTATT	GCACACGTTTGCCACTCATC

Supplementary Table S2. The phylogentic tree was constructed using 258, 83, 45 protein sequences in the wheat, rice, and Arabidopsis genome, respectively.

Triticum aestivum (wheat)

Name	Gene ID	Name	Gene ID
bZIP_1	TraesCSU02G019200.1 248 -310	TraesCSU02G019200.1 248 -310	Grp G
bZIP_2	TraesCS5D02G415300.1 63 - 113	TraesCS5D02G415300.1 63 - 113	Grp S
bZIP_3	TraesCS5D02G473000.2 255 -318	TraesCS5D02G473000.2 255 -318	Grp G
bZIP_4	TraesCS6A02G087400.1 220 -282	TraesCS6A02G087400.1 220 -282	Grp G
bZIP_5	TraesCS6A02G087400.2 251 -313	TraesCS6A02G087400.2 251 -313	Grp G
bZIP_6	TraesCS5B02G410000.1 62 - 114	TraesCS5B02G410000.1 62 - 114	Grp S
bZIP_7	TraesCS5B02G470600.1 251 -314	TraesCS5B02G470600.1 251 -314	Grp G
bZIP_8	TraesCS4A02G198100.1 127 -189	TraesCS4A02G198100.1 127 -189	Grp H
bZIP_9	TraesCS2A02G245700.1 258 -320	TraesCS2A02G245700.1 258 -320	Grp G
bZIP_10	TraesCS1B02G439800.1 263 -325	TraesCS1B02G439800.1 263 -325	Grp G
bZIP_11	TraesCS1D02G417100.1 263 -325	TraesCS1D02G417100.1 263 -325	Grp G
bZIP_12	TraesCS5D02G124600.1 291 -353	TraesCS5D02G124600.1 291 -353	Grp G
bZIP_13	TraesCS4D02G230200.1 280 -342	TraesCS4D02G230200.1 280 -342	Grp G
bZIP_14	TraesCS5D02G134100.1 114 -176	TraesCS5D02G134100.1 114 -176	Grp H
bZIP_15	TraesCS6B02G110100.1 250 -312	TraesCS6B02G110100.1 250 -312	Grp G
bZIP_16	TraesCS2B02G269600.1 259 -321	TraesCS2B02G269600.1 259 -321	Grp G
bZIP_17	TraesCS5A02G460800.1 249 -312	TraesCS5A02G460800.1 249 -312	Grp G
bZIP_18	TraesCS4B02G117400.1 128 -190	TraesCS4B02G117400.1 128 -190	Grp H
bZIP_19	TraesCS4B02G117400.2 221 -283	TraesCS4B02G117400.2 221 -283	Grp H
bZIP_20	TraesCS5A02G126600.1 114 -176	TraesCS5A02G126600.1 114 -176	Grp G
bZIP_21	TraesCS5B02G122000.1 269 -331	TraesCS5B02G122000.1 269 -331	Grp G
bZIP_22	TraesCS5B02G125800.1 114 -176	TraesCS5B02G125800.1 114 -176	Grp G
bZIP_23	TraesCS2D02G236200.1 258 -320	TraesCS2D02G236200.1 258 -320	Grp G
bZIP_24	TraesCS4B02G229100.2 323 -385	TraesCS4B02G229100.2 323 -385	Grp G
bZIP_25	TraesCS7D02G349300.1 128 -189	TraesCS7D02G349300.1 128 -189	GrpB
bZIP_26	TraesCS5D02G258000.1 89 - 144	TraesCS5D02G258000.1 89 - 144	Grp S
bZIP_27	TraesCS5D02G273500.1 206 -237	TraesCS6A02G175800.1 81 - 142	GrpB
bZIP_28	TraesCS6A02G175800.1 81 - 142	TraesCS7D02G182900.1 105 -154	Grp S
bZIP_29	TraesCS7D02G182900.1 105 -154	TraesCS7A02G181100.1 106 -156	Grp S
bZIP_30	TraesCS7A02G181100.1 106 -156	TraesCS7A02G373800.1 83 - 144	GrpB
bZIP_31	TraesCS7A02G373800.1 83 - 144	TraesCS6B02G209600.1 81 - 142	Grp H
bZIP_32	TraesCS6B02G209600.1 81 - 142	TraesCS7B02G086100.1 105 -154	Grp S
bZIP_33	TraesCS7B02G086100.1 105 -154	TraesCS3A02G128900.1 88 - 149	Grp H
bZIP_34	TraesCS3A02G128900.1 88 - 149	TraesCS5A02G250400.1 90 - 145	Grp S
bZIP_35	TraesCS5A02G250400.1 90 - 145	TraesCS6D02G167800.1 81 - 142	Grp H
bZIP_36	TraesCS5A02G265600.1 207 -238	TraesCS5B02G248400.1 90 - 145	Grp S
bZIP_37	TraesCS6D02G167800.1 81 - 142	TraesCS7B02G293900.1 72 - 122	Grp S
bZIP_38	TraesCS5B02G248400.1 90 - 145	TraesCS6D02G270000.1 103 -160	Grp S
bZIP_39	TraesCS5B02G265300.1 214 -245	TraesCS7D02G213200.1 86 - 134	Grp S
bZIP_40	TraesCS5B02G265300.2 214 -245	TraesCS6A02G288900.1 101 -152	Grp S
bZIP_41	TraesCS7B02G293900.1 72 - 122	TraesCS1B02G268500.1 174 -234	GrpB
bZIP_42	TraesCS6D02G270000.1 103 -160	TraesCS1B02G282800.1 170 -229	Grp A

bZIP_43	TraesCS7D02G213200.1 86 - 134	TraesCS1D02G257400.1 177 -237	GrpB
bZIP_44	TraesCS6A02G288900.1 101 -152	TraesCS2A02G142800.1 125 -185	Grp A
bZIP_45	TraesCS1B02G268500.1 174 -234	TraesCS1D02G273100.1 170 -229	Grp A
bZIP_46	TraesCS1B02G282800.1 170 -229	TraesCS7A02G211500.1 87 - 135	Grp S
bZIP_47	TraesCS1D02G257400.1 177 -237	TraesCS3A02G330800.1 70 - 118	Grp F
bZIP_48	TraesCS2A02G142800.1 125 -185	TraesCS5D02G068800.1 106 -160	Grp C
bZIP_49	TraesCS1D02G273100.1 170 -229	TraesCS3A02G378700.1 175 -235	Grp A
bZIP_50	TraesCS7A02G211500.1 87 - 135	TraesCS2B02G167900.1 122 -182	GrpB
bZIP_51	TraesCS3A02G330800.1 70 - 118	TraesCS7A02G391800.1 147 -198	Grp S
bZIP_52	TraesCS5D02G068800.1 106 -160	TraesCS5D02G150700.1 241 -289	Grp A
bZIP_53	TraesCS3A02G378700.1 175 -235	TraesCS5D02G150700.2 241 -287	Grp A
bZIP_54	TraesCS2B02G167900.1 122 -182	TraesCS1A02G273000.2 117 -176	Grp A
bZIP_55	TraesCS7A02G391800.1 147 -198	TraesCS6B02G318700.1 104 -161	Grp S
bZIP_56	TraesCS5D02G150700.1 241 -289	TraesCS5A02G057500.1 106 -160	Grp C
bZIP_57	TraesCS5D02G150700.2 241 -287	TraesCS2D02G146100.1 123 -183	Grp A
bZIP_58	TraesCS1A02G273000.2 117 -176	TraesCS5B02G059200.1 106 -160	Grp C
bZIP_59	TraesCS6B02G318700.1 104 -161	TraesCS7B02G118500.1 86 - 134	Grp S
bZIP_60	TraesCS5A02G057500.1 106 -160	TraesCS5A02G141500.1 241 -287	Grp A
bZIP_61	TraesCS2D02G146100.1 123 -183	TraesCS5B02G139800.1 241 -287	Grp A
bZIP_62	TraesCS5B02G059200.1 106 -160	TraesCS3D02G324200.1 71 - 119	Grp F
bZIP_63	TraesCS7B02G118500.1 86 - 134	TraesCS3D02G371900.1 168 -228	Grp A
bZIP_64	TraesCS5A02G141500.1 241 -287	TraesCS7D02G475100.1 143 -194	Grp C
bZIP_65	TraesCS5B02G139800.1 241 -287	TraesCS5D02G338000.1 129 -182	Grp A
bZIP_66	TraesCS3D02G324200.1 71 - 119	TraesCS7B02G391800.1 144 -195	Grp C
bZIP_67	TraesCS3D02G371900.1 168 -228	TraesCS7B02G464700.1 94 - 149	Grp A
bZIP_68	TraesCS7D02G475100.1 143 -194	TraesCS6A02G154600.1 137 -188	Grp C
bZIP_69	TraesCS5D02G338000.1 129 -182	TraesCS5B02G332300.1 129 -182	Grp A
bZIP_70	TraesCS7B02G391800.1 144 -195	TraesCS4A02G291200.1 55 - 102	Grp S
bZIP_71	TraesCS7B02G464700.1 94 - 149	TraesCS5A02G332000.1 134 -187	Grp A
bZIP_72	TraesCS6A02G154600.1 137 -188	TraesCS7A02G488600.1 146 -197	Grp C
bZIP_73	TraesCS6A02G165800.1 160 -200	TraesCS6B02G182500.1 136 -187	Grp C
bZIP_74	TraesCS5B02G332300.1 129 -182	TraesCS2D02G536700.1 106 -150	Grp A
bZIP_75	TraesCS4A02G291200.1 55 - 102	TraesCS6D02G144400.1 135 -186	Grp C
bZIP_76	TraesCS3A02G372400.3 244 -279	TraesCS7D02G268400.1 174 -232	Grp I
bZIP_77	TraesCS5A02G332000.1 134 -187	TraesCS7D02G269300.1 318 -367	Grp I
bZIP_78	TraesCS2B02G556600.1 133 -173	TraesCS7D02G392800.1 143 -199	GrpB
bZIP_79	TraesCS7A02G488600.1 146 -197	TraesCS5D02G244500.2 274 -324	Grp A
bZIP_80	TraesCS6B02G182500.1 136 -187	TraesCS7B02G299200.1 143 -199	GrpB
bZIP_81	TraesCS6B02G193200.1 151 -182	TraesCS5D02G308600.1 177 -235	Grp I
bZIP_82	TraesCS2D02G529000.1 124 -164	TraesCS5D02G315400.1 329 -383	Grp E
bZIP_83	TraesCS2D02G536700.1 106 -150	TraesCS5D02G447500.1 219 -269	Grp C
bZIP_84	TraesCS2A02G526300.1 122 -162	TraesCS6D02G312800.1 281 -326	Grp A
bZIP_85	TraesCS3D02G365200.2 251 -286	TraesCS6A02G170600.1 82 - 128	Grp S
bZIP_86	TraesCS6D02G144400.1 135 -186	TraesCS5B02G301900.1 177 -235	Grp I
bZIP_87	TraesCS6D02G154400.1 160 -200	TraesCS5B02G308800.1 329 -383	Grp E
bZIP_88	TraesCS7D02G268400.1 174 -232	TraesCS1B02G193800.1 243 -298	Grp I
bZIP_89	TraesCS7D02G269300.1 318 -367	TraesCS7D02G171300.1 265 -316	Grp A
bZIP_90	TraesCS7D02G392800.1 143 -199	TraesCS4A02G128900.1 155 -197	Grp A

bZIP_91	TraesCS5D02G244500.2 274 -324	TraesCS4A02G135600.2 160 -214	Grp I
bZIP_92	TraesCS7B02G299200.1 143 -199	TraesCS1D02G191000.1 242 -297	Grp I
bZIP_93	TraesCS5D02G308600.1 177 -235	TraesCS5B02G444100.2 212 -262	Grp C
bZIP_94	TraesCS5D02G315400.1 329 -383	TraesCS4A02G183400.1 234 -270	Grp F
bZIP_95	TraesCS5D02G447500.1 219 -269	TraesCS1B02G285800.2 215 -243	Grp D
bZIP_96	TraesCS6D02G312800.1 281 -326	TraesCS6A02G333600.1 271 -316	Grp A
bZIP_97	TraesCS6A02G170600.1 82 - 128	TraesCS7A02G170600.1 263 -314	Grp A
bZIP_98	TraesCS5B02G301900.1 177 -235	TraesCS4A02G209900.1 168 -222	Grp E
bZIP_99	TraesCS5B02G308800.1 329 -383	TraesCS1B02G317100.1 252 -295	Grp A
bZIP_100	TraesCS1B02G193800.1 243 -298	TraesCS1D02G276100.1 215 -243	Grp D
bZIP_101	TraesCS7D02G171300.1 265 -316	TraesCS3A02G293700.1 139 -184	Grp E
bZIP_102	TraesCS4A02G128900.1 155 -197	TraesCS7A02G207100.1 159 -200	Grp F
bZIP_103	TraesCS4A02G135600.2 160 -214	TraesCS1B02G343500.1 211 -256	Grp C
bZIP_104	TraesCS1D02G191000.1 242 -297	TraesCS1D02G306000.1 257 -300	Grp A
bZIP_105	TraesCS5B02G444100.2 212 -262	TraesCS3A02G334100.1 190 -230	Grp F
bZIP_106	TraesCS4A02G183400.1 234 -270	TraesCS2B02G115700.1 179 -225	Grp A
bZIP_107	TraesCS1B02G285800.2 215 -243	TraesCS1D02G332200.1 206 -252	Grp C
bZIP_108	TraesCS6A02G333600.1 271 -316	TraesCS2B02G122700.1 165 -209	Grp I
bZIP_109	TraesCS7A02G170600.1 263 -314	TraesCS7A02G266400.1 171 -229	Grp I
bZIP_110	TraesCS4A02G209900.1 168 -222	TraesCS7A02G268700.1 318 -367	Grp I
bZIP_111	TraesCS1B02G317100.1 252 -295	TraesCS3A02G371800.1 307 -355	Grp A
bZIP_112	TraesCS1D02G276100.1 215 -243	TraesCS3A02G372000.1 298 -344	Grp A
bZIP_113	TraesCS3A02G293700.1 139 -184	TraesCS3A02G372200.1 298 -345	Grp A
bZIP_114	TraesCS7A02G207100.1 159 -200	TraesCS3A02G372400.2 261 -295	Grp S
bZIP_115	TraesCS1B02G343500.1 211 -256	TraesCS2B02G165700.1 177 -227	Grp D
bZIP_116	TraesCS1D02G306000.1 257 -300	TraesCS7A02G398400.1 143 -199	GrpB
bZIP_117	TraesCS3A02G334100.1 190 -230	TraesCS5D02G132300.1 163 -221	Grp E
bZIP_118	TraesCS2B02G115700.1 179 -225	TraesCS2B02G523600.1 95 - 140	Grp A
bZIP_119	TraesCS1D02G332200.1 206 -252	TraesCS5D02G148700.1 112 -160	Grp E
bZIP_120	TraesCS2B02G122700.1 165 -209	TraesCS1A02G186000.1 246 -301	Grp I
bZIP_121	TraesCS7A02G266400.1 171 -229	TraesCS2B02G564900.1 106 -149	Grp A
bZIP_122	TraesCS7A02G268700.1 318 -367	TraesCS2A02G352100.1 168 -224	Grp I
bZIP_123	TraesCS3A02G371800.1 307 -355	TraesCS5A02G440400.1 204 -254	Grp C
bZIP_124	TraesCS3A02G372000.1 298 -344	TraesCS4D02G316900.1 110 -164	Grp I
bZIP_125	TraesCS3A02G372200.1 298 -345	TraesCS6B02G198700.1 82 - 128	Grp S
bZIP_126	TraesCS3A02G372400.2 261 -295	TraesCS2A02G099400.1 179 -225	Grp A
bZIP_127	TraesCS2B02G165700.1 177 -227	TraesCS3D02G148200.1 175 -225	Grp D
bZIP_128	TraesCS7A02G398400.1 143 -199	TraesCS2A02G105700.1 163 -207	Grp I
bZIP_129	TraesCS5D02G132300.1 163 -221	TraesCS5A02G491600.1 102 -156	Grp I
bZIP_130	TraesCS2B02G523600.1 95 - 140	TraesCS1A02G306300.1 254 -297	Grp A
bZIP_131	TraesCS5D02G148700.1 112 -160	TraesCS2D02G495700.1 111 -156	Grp A
bZIP_132	TraesCS1A02G186000.1 246 -301	TraesCS1A02G329900.1 211 -249	Grp C
bZIP_133	TraesCS2B02G564900.1 106 -149	TraesCS2D02G098700.1 179 -225	Grp A
bZIP_134	TraesCS5D02G178800.1 179 -207	TraesCS2D02G105400.1 163 -207	Grp E
bZIP_135	TraesCS2A02G352100.1 168 -224	TraesCS2D02G105400.2 163 -207	Grp I
bZIP_136	TraesCS5A02G440400.1 204 -254	TraesCS6B02G364000.1 272 -317	Grp A
bZIP_137	TraesCS4D02G316900.1 110 -164	TraesCS2D02G536800.1 109 -152	Grp A
bZIP_138	TraesCS6B02G198700.1 82 - 128	TraesCS2A02G495400.1 111 -156	Grp A

bZIP_139	TraesCS2A02G099400.1	179 -225	TraesCS7B02G075600.1	260 -311	Grp A
bZIP_140	TraesCS3D02G148200.1	175 -225	TraesCS2D02G144300.1	177 -227	Grp D
bZIP_141	TraesCS2A02G105700.1	163 -207	TraesCS2A02G534200.1	107 -150	Grp A
bZIP_142	TraesCS1A02G276600.1	215 -243	TraesCS2A02G534300.1	94 - 137	Grp A
bZIP_143	TraesCS5A02G491600.1	102 -156	TraesCS4B02G113400.1	168 -222	Grp E
bZIP_144	TraesCS1A02G306300.1	254 -297	TraesCS7B02G114300.1	164 -205	Grp D
bZIP_145	TraesCS2D02G495700.1	111 -156	TraesCS3B02G167500.1	163 -212	Grp D
bZIP_146	TraesCS1A02G329900.1	211 -249	TraesCS5A02G124600.1	163 -221	Grp E
bZIP_147	TraesCS2D02G098700.1	179 -225	TraesCS5A02G143300.1	112 -160	Grp E
bZIP_148	TraesCS2D02G105400.1	163 -207	TraesCS4B02G169600.1	160 -214	Grp I
bZIP_149	TraesCS2D02G105400.2	163 -207	TraesCS3D02G293500.1	144 -189	Grp D
bZIP_150	TraesCS6B02G364000.1	272 -317	TraesCS7B02G166100.1	175 -233	Grp I
bZIP_151	TraesCS2D02G536800.1	109 -152	TraesCS4B02G175800.1	155 -197	Grp A
bZIP_152	TraesCS2A02G495400.1	111 -156	TraesCS5B02G124200.1	163 -221	Grp E
bZIP_153	TraesCS7B02G075600.1	260 -311	TraesCS5B02G142200.1	112 -160	Grp D
bZIP_154	TraesCS2D02G144300.1	177 -227	TraesCS3D02G327600.1	192 -232	Grp D
bZIP_155	TraesCS2A02G534200.1	107 -150	TraesCS3D02G330300.1	267 -311	Grp A
bZIP_156	TraesCS2A02G534300.1	94 - 137	TraesCS3A02G131900.1	175 -209	Grp D
bZIP_157	TraesCS4B02G113400.1	168 -222	TraesCS5A02G237200.2	289 -335	Grp A
bZIP_158	TraesCS7B02G114300.1	164 -205	TraesCS3D02G364900.1	306 -354	Grp A
bZIP_159	TraesCS3B02G167500.1	163 -212	TraesCS5A02G299400.1	177 -235	Grp I
bZIP_160	TraesCS5A02G124600.1	163 -221	TraesCS4B02G320400.1	245 -299	Grp I
bZIP_161	TraesCS4B02G135000.1	260 -296	TraesCS5A02G308400.1	372 -426	Grp E
bZIP_162	TraesCS5A02G143300.1	112 -160	TraesCS6D02G160200.1	81 - 127	Grp S
bZIP_163	TraesCS4B02G169600.1	160 -214	TraesCS5B02G235600.1	287 -337	Grp A
bZIP_164	TraesCS3D02G293500.1	144 -189	TraesCS7D02G527500.1	95 - 148	Grp A
bZIP_165	TraesCS7B02G166100.1	175 -233	TraesCS1B02G323600.1	74 - 119	Grp F
bZIP_166	TraesCS4B02G175800.1	155 -197	TraesCS1D02G312200.1	74 - 119	Grp D
bZIP_167	TraesCS5B02G124200.1	163 -221	TraesCS3A02G371900.1	299 -345	Grp A
bZIP_168	TraesCS5A02G174200.1	180 -208	TraesCS4D02G335000.1	109 -158	Grp A
bZIP_169	TraesCS5B02G142200.1	112 -160	TraesCS7A02G542700.1	95 - 148	Grp A
bZIP_170	TraesCS3D02G327600.1	192 -232	TraesCS5A02G508600.1	109 -158	Grp A
bZIP_171	TraesCS3D02G330300.1	267 -311	TraesCS1A02G311900.1	73 - 118	Grp D
bZIP_172	TraesCS3A02G131900.1	175 -209	TraesCS5B02G017700.1	88 - 134	Grp D
bZIP_173	TraesCS5A02G237200.2	289 -335	TraesCS2B02G489900.1	60 - 115	Grp A
bZIP_174	TraesCS3D02G364900.1	306 -354	TraesCS4B02G338700.1	109 -158	Grp A
bZIP_175	TraesCS3D02G365200.3	268 -302	TraesCS2A02G140900.1	179 -227	Grp D
bZIP_176	TraesCS3D02G365200.5	265 -299	TraesCS5D02G025800.1	90 - 135	Grp D
bZIP_177	TraesCS5A02G299400.1	177 -235	TraesCS5A02G020700.1	117 -161	Grp D
bZIP_178	TraesCS4B02G320400.1	245 -299	TraesCS3D02G365500.1	301 -347	Grp A
bZIP_179	TraesCS5A02G308400.1	372 -426	TraesCS7D02G518100.1	93 - 136	Grp D
bZIP_180	TraesCS6D02G160200.1	81 - 127	TraesCS7B02G447900.1	98 - 141	Grp S
bZIP_181	TraesCS5B02G235600.1	287 -337	TraesCS7A02G420400.1	158 -201	Grp S
bZIP_182	TraesCS7D02G527500.1	95 - 148	TraesCS7A02G530300.1	96 - 139	Grp F
bZIP_183	TraesCS1B02G323600.1	74 - 119	TraesCS3D02G365000.1	315 -359	Grp A
bZIP_184	TraesCS1D02G312200.1	74 - 119	TraesCS5D02G447500.2	160 -206	Grp C
bZIP_185	TraesCS3A02G371900.1	299 -345	TraesCS5B02G444100.1	159 -205	Grp C
bZIP_186	TraesCS4D02G335000.1	109 -158	TraesCS3A02G293700.2	178 -219	Grp D

bZIP_187	TraesCS7A02G542700.1 95 - 148	TraesCS5A02G440400.2 153 -199	Grp C
bZIP_188	TraesCS5A02G508600.1 109 -158	TraesCS5A02G405200.1 48 - 98	Grp S
bZIP_189	TraesCS1A02G311900.1 73 - 118	TraesCS4A02G120500.1 23 - 83	Grp S
bZIP_190	TraesCS5B02G017700.1 88 - 134	TraesCS5D02G183500.1 30 - 78	Grp S
bZIP_191	TraesCS2B02G489900.1 60 - 115	TraesCS4B02G184500.1 23 - 83	Grp S
bZIP_192	TraesCS4B02G338700.1 109 -158	TraesCS5A02G179000.1 30 - 78	Grp S
bZIP_193	TraesCS2A02G140900.1 179 -227	TraesCS5B02G176700.1 30 - 80	Grp C
bZIP_194	TraesCS5D02G025800.1 90 - 135	TraesCS6A02G096300.1 37 - 78	Grp S
bZIP_195	TraesCS5A02G020700.1 117 -161	TraesCS6B02G124700.1 38 - 79	Grp S
bZIP_196	TraesCS3D02G365500.1 301 -347	TraesCS3D02G203300.1 32 - 85	Grp S
bZIP_197	TraesCS7D02G518100.1 93 - 136	TraesCS3B02G226200.1 27 - 79	Grp S
bZIP_198	TraesCS7B02G447900.1 98 - 141	TraesCS6D02G087400.1 39 - 80	Grp S
bZIP_199	TraesCS7A02G420400.1 158 -201	TraesCS1B02G091500.1 28 - 81	Grp S
bZIP_200	TraesCS7A02G530300.1 96 - 139	TraesCS1D02G075500.1 28 - 81	Grp S
bZIP_201	TraesCS3D02G365000.1 315 -359	TraesCS1B02G127400.1 48 - 77	Grp S
bZIP_202	TraesCS5D02G447500.2 160 -206	TraesCS1D02G105300.1 48 - 86	Grp S
bZIP_203	TraesCS5B02G444100.1 159 -205	TraesCS4A02G126300.1 50 - 91	Grp D
bZIP_204	TraesCS3A02G293700.2 178 -219	TraesCS1A02G072600.1 28 - 81	Grp S
bZIP_205	TraesCS5A02G440400.2 153 -199	TraesCS2B02G113200.2 51 - 92	Grp F
bZIP_206	TraesCS5A02G405200.1 48 - 98	TraesCS1A02G096300.1 49 - 78	Grp S
bZIP_207	TraesCS4A02G120500.1 23 - 83	TraesCS5D02G100700.1 30 - 78	Grp S
bZIP_208	TraesCS5D02G183500.1 30 - 78	TraesCS4D02G316900.2 19 - 66	Grp I
bZIP_209	TraesCS4B02G184500.1 23 - 83	TraesCS2A02G097500.1 48 - 89	Grp F
bZIP_210	TraesCS5A02G179000.1 30 - 78	TraesCS3D02G194800.1 46 - 86	Grp D
bZIP_211	TraesCS5B02G176700.1 30 - 80	TraesCS2D02G096800.1 48 - 89	Grp F
bZIP_212	TraesCS6A02G096300.1 37 - 78	TraesCS5A02G088300.1 30 - 78	Grp S
bZIP_213	TraesCS6B02G124700.1 38 - 79	TraesCS5B02G094300.1 30 - 78	Grp S
bZIP_214	TraesCS3D02G203300.1 32 - 85	TraesCS3B02G220400.1 46 - 86	Grp D
bZIP_215	TraesCS3B02G226200.1 27 - 79	TraesCS4B02G178600.1 50 - 91	Grp D
bZIP_216	TraesCS6D02G087400.1 39 - 80	TraesCS3A02G190700.1 46 - 86	Grp D
bZIP_217	TraesCS1B02G091500.1 28 - 81	TraesCS3A02G200600.1 33 - 79	Grp S
bZIP_218	TraesCS1D02G075500.1 28 - 81	TraesCS4A02G126300.2 53 - 92	Grp D
bZIP_219	TraesCS1B02G127400.1 48 - 77	TraesCS4D02G115200.1 127- 189	Grp G
bZIP_220	TraesCS1D02G105300.1 48 - 86	TraesCS3D02G129800.1 88 - 149	Grp H
bZIP_221	TraesCS4A02G126300.1 50 - 91	TraesCS4D02G185500.1 23 - 83	Grp S
bZIP_222	TraesCS1A02G072600.1 28 - 81	TraesCS3B02G360900.1 109- 157	Grp D
bZIP_223	TraesCS2B02G113200.2 51 - 92	TraesCS3B02G411300.1 168- 227	Grp A
bZIP_224	TraesCS1A02G096300.1 49 - 78	TraesCS4D02G020600.1 55 - 99	Grp C
bZIP_225	TraesCS5D02G100700.1 30 - 78	TraesCS4D02G110900.1 167- 221	Grp E
bZIP_226	TraesCS4D02G316900.2 19 - 66	TraesCS4D02G171700.2 160- 214	Grp I
bZIP_227	TraesCS2A02G097500.1 48 - 89	TraesCS4D02G177700.1 153- 195	Grp A
bZIP_228	TraesCS3D02G194800.1 46 - 86	TraesCS4D02G180200.1 50 - 91	Grp D
bZIP_229	TraesCS2D02G096800.1 48 - 89	TraesCS3B02G328400.1 144- 189	Grp D
bZIP_230	TraesCS5A02G088300.1 30 - 78	TraesCS3B02G365100.1 192- 232	Grp D
bZIP_231	TraesCS5B02G094300.1 30 - 78	TraesCS3B02G368300.1 273- 317	Grp A
bZIP_232	TraesCS3B02G220400.1 46 - 86	TraesCS3B02G404200.1 306- 362	Grp A
bZIP_233	TraesCS4B02G178600.1 50 - 91	TraesCS3B02G404300.1 307- 355	Grp A
bZIP_234	TraesCS3A02G190700.1 46 - 86	TraesCS3B02G404400.1 307- 351	Grp A

bZIP_235	TraesCS3A02G200600.1	33 - 79
bZIP_236	TraesCS4A02G126300.2	53 - 92
bZIP_237	TraesCS4D02G115200.1	127- 189
bZIP_238	TraesCS3D02G129800.1	88 - 149
bZIP_239	TraesCS4D02G185500.1	23 - 83
bZIP_240	TraesCS3B02G360900.1	109- 157
bZIP_241	TraesCS3B02G411300.1	168- 227
bZIP_242	TraesCS3B02G404800.4	244- 279
bZIP_243	TraesCS4D02G020600.1	55 - 99
bZIP_244	TraesCS4D02G110900.1	167- 221
bZIP_245	TraesCS4D02G129900.1	232- 268
bZIP_246	TraesCS4D02G171700.2	160- 214
bZIP_247	TraesCS4D02G177700.1	153- 195
bZIP_248	TraesCS4D02G180200.1	50 - 91
bZIP_249	TraesCS3B02G328400.1	144- 189
bZIP_250	TraesCS3B02G365100.1	192- 232
bZIP_251	TraesCS3B02G368300.1	273- 317
bZIP_252	TraesCS3B02G404200.1	306- 362
bZIP_253	TraesCS3B02G404300.1	307- 355
bZIP_254	TraesCS3B02G404400.1	307- 351
bZIP_255	TraesCS3B02G404600.1	307- 354
bZIP_256	TraesCS3B02G404800.2	261- 295
bZIP_257	TraesCS3B02G404500.1	308- 351
bZIP_258	TraesCS4D02G180200.4	53 - 92

TraesCS3B02G404600.1 307- 354	Grp A
TraesCS3B02G404500.1 308- 351	Grp A
TraesCS4D02G180200.4 53 - 92	Grp D

Oryza sativa (rice)

**Gene ID**	**Group**
LOC_Os02g03580 244-306	GrpG
LOC_Os02g58670 67-127	GrpA
LOC_Os03g13614 299-361	GrpG
LOC_Os03g56010 65-114	GrpS
LOC_Os03g59460 200-226	GrpG
LOC_Os05g49420 245-307	GrpG
LOC_Os07g03220 72-122	GrpS
LOC_Os07g10890 265-327	GrpG
LOC_Os11g05640 124-186	GrpG
LOC_Os12g13170 294-356	GrpG
LOC_Os12g43790 60-108	GrpS
LOC_Os01g07880 109-170	GrpH
LOC_Os01g46970 224-285	GrpG
LOC_Os02g10860 172-233	GrpH
LOC_Os03g19370 22-71	GrpS
LOC_Os06g39960 104-163	GrpH
LOC_Os07g08420 235-289	GrpC
LOC_Os08g38020 119-178	GrpS
LOC_Os09g29820 76-131	GrpS
LOC_Os09g31390 201-243	GrpD

Arabidopsis thaliana (Arabidopsis)

**Gene ID**	**Group**
AT_Q9C5Q2_192_234	GrpA
AT_Q9LES3_227_270	GrpA
AT_Q84JK2_216_265	GrpA
AT_Q7PCC6_164_213	GrpA
AT_Q9M7Q5_313_369	GrpA
AT_Q9M7Q3_374_430	GrpA
AT_Q9M7Q2_353_405	GrpA
AT_Q9SJN0_357_405	GrpA
AT_Q8RYD6_249_301	GrpA
AT_P42777_186_247	GrpA
AT_Q9FMM7_294_344	GrpD
AT_Q9SI15_31_82	GrpS
AT_O65683_26_67	GrpS
AT_C0Z2L5_41_81	GrpS
AT_Q9LZP8_25_83	GrpS
AT_Q9FMC2_72_118	GrpS
AT_Q9CA46_46_100	GrpS
AT_Q9FUD3_120_166	GrpC
AT_O22763_215_269	GrpC
AT_Q9M1G6_230_283	GrpC

LOC_Os01g64730 184-242	GrpA
LOC_Os02g49560 78-132	GrpS
LOC_Os04g54474 104-144	GrpD
LOC_Os05g34050 172-232	GrpB
LOC_Os05g36160 186-237	GrpA
LOC_Os05g41540 69-117	GrpF
LOC_Os06g45140 134-186	GrpC
LOC_Os07g44950 111-171	GrpB
LOC_Os08g07970 107-142	GrpD
LOC_Os08g26880 36-95	GrpS
LOC_Os09g13570 29-77	GrpS
LOC_Os01g17260 44-85	GrpD
LOC_Os01g64020 228-259	GrpD
LOC_Os02g03960 36-78	GrpS
LOC_Os02g07840 123-172	GrpC
LOC_Os02g10140 134-174	GrpD
LOC_Os02g16680 142-191	GrpC
LOC_Os05g37170 230-260	GrpD
LOC_Os06g41770 210-267	GrpB
LOC_Os06g42690 69-128	GrpS
LOC_Os06g50480 96-143	GrpA
LOC_Os06g50600 96-152	GrpA
LOC_Os06g50830 96-152	GrpA
LOC_Os09g36910 125-178	GrpA
LOC_Os12g37410 25-84	GrpS
LOC_Os12g40920 115-169	GrpC
LOC_Os01g11350 190-240	GrpE
LOC_Os01g36220 42-99	GrpS
LOC_Os01g55150 139-184	GrpE
LOC_Os01g59350 187-222	GrpD
LOC_Os01g59760 265-308	GrpA
LOC_Os01g64000 304-350	GrpA
LOC_Os02g09830 74-120	GrpS
LOC_Os02g14910 142-192	GrpE
LOC_Os02g52780 277-327	GrpA
LOC_Os03g03550 277-335	GrpI
LOC_Os03g20310 49-79	GrpD
LOC_Os03g20650 162-203	GrpA
LOC_Os03g21800 134-180	GrpI
LOC_Os03g47200 52-93	GrpS
LOC_Os03g58250 234-285	GrpC
LOC_Os04g41820 270-328	GrpI
LOC_Os05g03860 25-79	GrpS
LOC_Os05g41070 265-308	GrpA
LOC_Os06g10880 244-296	GrpA
LOC_Os06g15480 95-135	GrpD
LOC_Os06g41100 149-177	GrpD
LOC_Os07g48180 175-220	GrpI

AT_B9DGI8_152_199	GrpC
AT_Q9LQ65_49_96	GrpS
AT_Q501B2_304_365	GrpG
AT_Q84LG2_293_355	GrpG
AT_P42774_220_282	GrpG
AT_P42775_247_309	GrpG
AT_P42776_257_319	GrpG
AT_P43273_46_86	GrpD
AT_Q39163_46_86	GrpD
AT_Q39140_46_86	GrpD
AT_Q93XM6_177_218	GrpD
AT_E3VNM4_161_191	GrpD
AT_Q9SX27_166_206	GrpD
AT_Q93ZE2_92_134	GrpD
AT_Q39237_82_113	GrpD
AT_Q39162_78_108	GrpD
AT_Q9M2K4_204_248	GrpE
AT_O22873_150_208	GrpD
AT_Q04088_203_255	GrpD
AT_Q9MA75_196_254	GrpD
AT_O24646_86_147	GrpH
AT_Q8W191_77_136	GrpF
AT_O22208_227_288	GrpB
AT_Q9LXX4_172_231	GrpB
AT_Q9SG86_192_249	GrpB

LOC_Os07g48660	183-225	GrpA
LOC_Os07g48820	50-91	GrpD
LOC_Os08g36790	243-295	GrpA
LOC_Os08g43090	322-380	GrpI
LOC_Os08g43600	72-122	GrpA
LOC_Os09g10840	189-218	GrpD
LOC_Os09g28310	297-347	GrpA
LOC_Os09g34060	183-241	GrpI
LOC_Os10g38820	248-306	GrpI
LOC_Os11g05480	180-209	GrpD
LOC_Os11g06170	162-209	GrpI
LOC_Os12g06520	164-221	GrpI
LOC_Os12g05680	184-212	GrpD
LOC_Os05g41280	24-63	GrpD
LOC_Os06g50310	97-142	GrpF

Supplementary Table S3. bZIP transcription factor proteins - proposed functionalities, processes and cellular components based on Gene Ontology results.

bZIP	Gene	Functional name	Molecular function	Biological process	Cellular component
ABF2	TraesCS5D02G244500	bZIP transcription factor TRAB1	transcription regulatory region sequence-specific DNA binding,<br>DNA-binding transcription factor activity	regulation of transcription, DNA-templated, abscisic acid-activated signalling pathway, positive regulation of transcription	nucleus
ABF4	TraesCS5A02G237200	bZIP transcription factor TRAB1	transcription regulatory region sequence-specific DNA binding<br>DNA-binding transcription factor activity	abscisic acid-activated signalling pathway<br>regulation of transcription, DNA-templated, positive regulation of transcription	nucleus
ABI5	TraesCS1A02G306300	ABSCISIC ACID-INSENSITIVE 5-like protein 2	DNA-binding transcription factor activity<br>DNA binding	positive regulation of transcription,<br>DNA-templated<br>response to abscisic acid,<br>abscisic acid-activated signalling pathway	nucleus
EMBP-1	TraesCS2B02G269600	DNA-binding protein EMBP-1	DNA-binding transcription factor activity,<br>sequence-specific DNA binding	regulation of transcription, DNA-templated abscisic acid-activated signalling pathway	nucleus
VIP1	TraesCS5B02G124200	transcription factor VIP1	nucleic acid binding<br>mitogen-activated protein kinase binding<br>chromatin binding<br>DNA-binding transcription factor activity<br>sequence-specific DNA binding	defence response, thigmotropism response to osmotic stress, osmosensory signalling pathway,<br>sulphate transport, DNA mediated transformation<br>import into nucleus, negative regulation of cell differentiation	nucleus cytosol

			protein self-association		
